# Hypernatremia at admission predicts poor survival in patients with terminal cancer: a retrospective cohort study

**DOI:** 10.1186/s12904-020-00607-z

**Published:** 2020-07-01

**Authors:** Min-Seok Seo, In Cheol Hwang, Jaehun Jung, Hwanhee Lee, Jae Hee Choi, Jae-Yong Shim

**Affiliations:** 1 Department of Family Medicine, Incheon St. Mary’s Hospital, 56 Dongsuro, Bupyung-gu, Incheon, Republic of Korea; 2 Department of Family Medicine, Yonsei University Graduate School of Medicine, 211 Eonju-ro, Dogok-dong, Gangnam-gu, Seoul, Republic of Korea; 3 Department of Family Medicine, Gil Medical Center, Gachon University College of Medicine, 1198 Guwol-dong, Namdong-gu, Incheon, 405-760 Republic of Korea; 4 Artificial Intelligence and Bigdata Convergence Center, Gachon University College of Medicine, Guwol-dong, Namdong-gu, Incheon, 405-760 Republic of Korea

**Keywords:** Electrolyte imbalance, Hypernatremia, Prognosis, Terminal cancer

## Abstract

**Background:**

Although palliative care providers, patients, and their families rely heavily on accurate prognostication, the prognostic value of electrolyte imbalance has received little attention.

**Methods:**

As a retrospective review, we screened inpatients with terminal cancer admitted between January 2017 and May 2019 to a single hospice-palliative care unit. Clinical characteristics and laboratory results were obtained from medical records for multivariable Cox regression analysis of independent prognostic factors.

**Results:**

Of the 487 patients who qualified, 15 (3%) were hypernatremic upon admission. The median survival time was 26 days. Parameters associated with shortened survival included male sex, advanced age (> 70 years), lung cancer, poor performance status, elevated inflammatory markers, azotemia, impaired liver function, and hypernatremia. In a multivariable Cox proportional hazards model, male sex (hazard ratio [HR] = 1.53, 95% confidence interval [CI]: 1.15–2.04), poor performance status (HR = 1.45, 95% CI: 1.09–1.94), leukocytosis (HR = 1.98, 95% CI: 1.47–2.66), hypoalbuminemia (HR = 2.06, 95% CI: 1.49–2.73), and hypernatremia (HR = 1.55, 95% CI: 1.18–2.03) emerged as significant predictors of poor prognosis.

**Conclusion:**

Hypernatremia may be a useful gauge of prognosis in patients with terminal cancer. Further large-scale prospective studies are needed to corroborate this finding.

## Background

Accurately predicting the prognosis of patients with terminal cancer is helpful to guide clinical decisions and develop palliative care management plans. Over the past few decades, a number of studies have been conducted in this setting to identify patient-related prognostic factors, including performance status, anorexia-cachexia, delirium, and dyspnea [1]; and various laboratory abnormalities have similarly been identified. Serum markers of systemic inflammation (i.e., leukocytosis, lymphopenia, C-reactive protein [CRP] elevation, and inflammatory cytokines) are well-known indices of poor survival [2]. Biomarkers of hepatic dysfunction (elevated lactate dehydrogenase [LDH], prolonged international normalized ratio [INR], hypoalbuminemia) and renal impairment (serum urea, creatinine, and uric acid elevations) have also been implicated [2, 3].

Electrolyte abnormalities are common among terminal cancer patients and represent yet another significant means of predicting survival [4]. In an earlier study, 79% of such patients showed at least one electrolyte abnormality upon referral for palliative care [5]. Hyponatremia is the most frequent electrolyte disorder of patients with terminal cancer, having dire consequences [6]. Its early detection and proper correction may actually prolong median survival time [7]. In cancer patients, hypernatremia caused by various etiologies such as excessive gain of sodium due to inadequate fluid replacement, excessive loss of free water, the use of osmotic agents, decreased release of antidiuretic hormone (ADH) and renal dysfunction to ADH [8]. Although hypernatremia is otherwise rare in these patients, offering little opportunity for study, there is evidence that the prognostic ramifications are negative due to a failure of feedback to compensate the imbalance [9, 10]. Potassium imbalance is also a highly prevalent electrolyte disorder. In cancer patients, hypokalemia often presents in conjunction with hyponatremia and hypomagnesemia [4]. Previous studies suggested that hyperkalemia is a prognostically unfavorable determinant, but this view remains controversial [11, 12]. Research on the prognostic utility of other electrolyte disorders including hypercalcemia and hypermagnesemia, has also been inconclusive [5, 13].

The present study was undertaken to assess the prevalence of electrolyte imbalance in terminally ill cancer patients and, investigate the potential prognostic significance.

## Methods

This retrospective study reviewed medical records of 515 patients admitted to the palliative care unit of Incheon regional cancer center between January 2017 and May 2019. All participants were terminally ill with cancer and were not expected to survive beyond 6 months based on the clinical assessment of a medical oncologist and surgeon [14]. Ultimately, 10 patients who lacked serum electrolyte data and 18 patients transferred from other medical institutions were excluded, leaving 487 patients eligible for the final analysis. Patients who had no survival information available due to discharge to home or other institutions were also censored (*n* = 19). The institutional review board of Gachon University Gil Medical Center approved this study (GCIRB2019–149), waiving the need for informed patient consent as designed.

Electronic medical records provided the following patient data: age, sex, primary cancer site, Eastern Cooperative Oncology Group Performance Status (ECOG-PS), evidence of active infection, survival times, and laboratory diagnostic results. The ECOG-PS is a scale (0–4) used to assess physical ability. On day of admission, an experienced member of the palliative care team (physicians and registered nurses) scored each patient’s functionality. Survival time was defined as the period from admission-day blood testing (electrolytes included) until the day of death. Laboratory testing on day of admission included total white blood cell (WBC) count with differential, hemoglobin, platelet count, serum creatinine, serum albumin, total bilirubin, INR, CRP, serum sodium, and potassium. Active infection was determined based on the use of antibiotics.

Descriptive data are expressed as medians and numbers of subjects. The Kaplan-Meier method and log-rank tests were invoked to measure differences in survival times across all patient characteristics, using Cox proportional hazards models to identify independent predictors of survival in univariable and multivariable analyses. Variables showing significance (*P* < 0.05) in univariable analysis were used in the final multivariable analysis. A two-tailed *P* < 0.05 was deemed significant. All computations were performed using standard software (Stata SE v9.2; StataCorp, College Station, TX, USA).

## Results

A total of 487 patients (men: 268, 55%; women: 219, 45%) with terminal cancer qualified for study participation. Baseline clinical characteristics of the study population are shown in Table 1. Median age was 70 years. The most common type of malignancy was cancer of the gastrointestinal tract (127 patients, 26.1%), followed by hepatopancreatobiliary cancer (118 patients, 24.2%). ECOG-PS scores of 3 and 4 were recorded in 38.8 and 37.6% of patients, respectively. The median values of abnormal laboratory parameters were as follows: hemoglobin 10.1 g/dL, CRP 5.2 mg/dL, and sodium 134 mEq/L. The median survival time overall was 26 days.

**Table 1:** Characteristics of study participants (*N* = 487)

	Median (IQR) or n (%)	Reference range
Age, yrs	70 (59–79)	
Female sex	219 (45.0)	
Cancer site		
Gastrointestinal tract	127 (26.1)	
Hepatobiliary/pancreatic	118 (24.2)	
Lung	108 (22.2)	
Urogenital tract	57 (11.7)	
Others	77 (15.8)	
ECOG-PS		
≤ 2	115 (23.6)	
3	189 (38.8)	
4	183 (37.6)	
Active infection^a^	164 (33.7)	
Laboratory parameter		
White blood cells, 10^3^/mm^3^	9.1 (6.3–12.7)	3.8–10
Neutrophils, %	78.9 (72.0–85.7)	50–75
Lymphocytes, %	11.2 (6.8–17.4)	20–44
Hemoglobin, g/dL	10.1 (8.8–11.7)	13–17
Platelets, 10^3^/mm^3^	227 (154–323)	150–400
Creatinine, mg/dL	0.8 (0.5–1.2)	0.5–1.2
Albumin, g/dL	3.2 (2.8–3.7)	3.5–5.2
Total bilirubin, mg/dL	0.7 (0.5–1.4)	0.2–1.2
PT/INR	1.2 (1.1–1.3)	0.8–1.2
C-reactive protein, mg/dL	5.2 (2.3–12.9)	0–0.5
Sodium, mEq/L	134 (130–137)	135–145
Potassium, mEq/L	4.2 (3.8–4.6)	3.5–5.5
Censored^b^	19 (3.9)	
Survival time, days	26 (14–45)	

*ECOG-PS* Eastern Cooperative Oncology Group performance status, *IQR* interquartile range, *PT/INR* prothrombin time/international normalized ratio

^a^Determined by the use of antibiotics

^b^No available information for survival by discharge or transfer

Table 2 shows the survival time according to participant characteristics. Advanced age (> 70 years), male sex, lung cancer, poor performance status, leukocytosis, neutrophilia, lymphopenia, thrombocytopenia, azotemia, hypoalbuminemia, hyperbilirubinemia, prolonged prothrombin time (PT)-INR, elevated CRP, and hypernatremia were associated with significantly shorter median survival time.

**Table 2:** Survival time in relation to patient characteristics

	n	Median survival, days (95% CI)	*P*^b^
Age, years			
< 70	232	29 (13–52)	**0.003**
≥ 70	255	24 (14–40)	
Sex			
Female	219	29 (13–48)	**0.035**
Male	268	24 (14–40)	
Cancer site			
Gastrointestinal tract	127	29 (14–47)	**0.012**
Hepatobiliary/pancreatic	118	24 (13–33)	
Lung	108	22 (12–41)	
Urogenital tract	57	27 (13–55)	
Others	77	35 (19–47)	
ECOG-PS			
≤ 2	115	30 (18–50)	**0.012**
3	189	27 (14–48)	
4	183	23 (11–40)	
Active infection			
No	322	27 (12–46)	0.558
Yes	164	25 (14–38)	
Leukocytosis			
No	279	31 (15–52)	**< 0.001**
Yes	208	22 (11–34)	
Neutrophilia			
No	173	31 (18–55)	**< 0.001**
Yes	314	24 (12–39)	
Lymphopenia			
No	90	36 (19–60)	**< 0.001**
Yes	397	25 (13–41)	
Anemia			
No	56	25 (13–45)	0.707
Yes	431	26 (14–45)	
Thrombocytopenia			
No	371	28 (14–47)	**0.020**
Yes	116	20 (11–36)	
Azotemia			
No	367	28 (14–46)	**0.011**
Yes	120	20 (12–38)	
Hypoalbuminemia			
No	176	35 (20–60)	**< 0.001**
Yes	311	20 (11–37)	
Hyperbilirubinemia			
No	341	29 (15–48)	**< 0.001**
Yes	145	19 (10–32)	
PT/INR prolongation			
No	259	29 (16–51)	**< 0.001**
Yes	200	20 (10–34)	
C-reactive protein^a^			
Low	239	29 (14–52)	**0.007**
High	233	22 (12–38)	
Sodium level			
Within normal range	219	28 (14–49)	**< 0.001**
Hyponatremia	253	25 (13–43)	
Hypernatremia	15	6 (3–28)	
Potassium level			
Within normal range	394	27 (14–46)	0.118
Hypokalemia	65	26 (13–43)	
Hyperkalemia	28	19 (10–27)	

*CI* confidence interval, *ECOG-PS* Eastern Cooperative Oncology Group performance status, *PT/INR* prothrombin time/international normalized ratio

^a^Median value applied

^b^Log-rank test

**Fig. 1:**
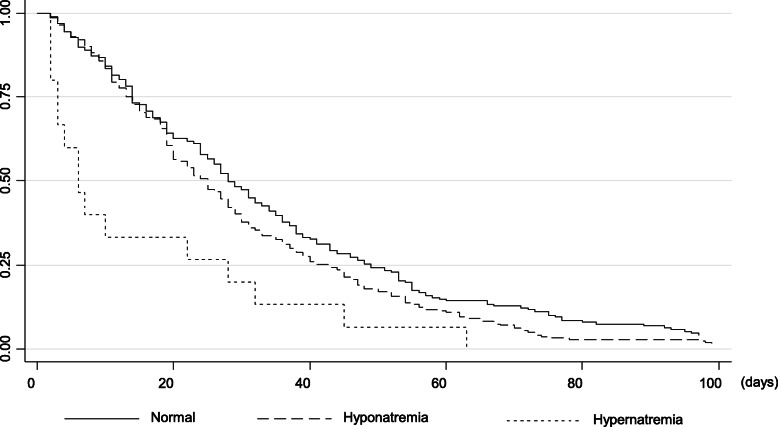
Kaplan-Meier survival curves of terminal cancer patients plotted by sodium level (note significantly shorter survival in patients with hypernatremia)

Kaplan-Meier survival curves plotted by serum sodium level are depicted in Fig. 1. Hypernatremia patients survived shorter than those with eunatremia or hyponatremia (*P* < 0.001), whereas the difference in survival time between eunatremic and hyponatremic patients was not significant.

Table 3 presents independent prognostic factors identified from the Cox proportional hazards models. Multivariable analysis revealed that various parameters including male sex (HR = 1.53; *P* = 0.004), poor ECOG-PS (HR = 1.45, *P* = 0.011), leukocytosis (HR = 1.98, *P* < 0.001), hypoalbuminemia (HR = 2.06, *P* < 0.001), and hypernatremia (HR = 1.55, *P* = 0.002), were significantly associated with poor survival.

**Table 3:** Independent prognostic indices of survival (Cox proportional hazards model)

	Univariable analysis		Multivariable analysis^a^	
	HR (95% CI)	*P*-value	HR (95% CI)	*P*-value
Advanced age (> 70 years)	1.31 (1.09–1.58)	**0.004**		
Male sex	1.21 (1.01–1.46)	**0.038**	1.53 (1.15–2.04)	0.004
Poor functional score (ECOG = 4)	1.30 (1.08–1.57)	**0.005**	1.45 (1.09–1.94)	0.011
Lung cancer	1.20 (0.96–1.50)	0.108		
Active infection	1.06 (0.87–1.28)	0.564		
Leukocytosis	1.56 (1.29–1.87)	**< 0.001**	1.98 (1.47–2.66)	< 0.001
Neutrophilia	1.45 (1.19–1.75)	**< 0.001**		
Lymphopenia	1.53 (1.20–1.94)	**0.001**		
Anemia	0.95 (0.71–1.26)	0.711		
Thrombocytopenia	1.28 (1.04–1.59)	**0.022**		
Azotemia	1.30 (1.06–1.61)	**0.013**		
Hypoalbuminemia	1.88 (1.54–2.28)	**< 0.001**	2.06 (1.49–2.73)	< 0.001
Hyperbilirubinemia	1.47 (1.21–1.80)	**< 0.001**		
PT/INR prolongation	1.46 (1.21–1.77)	**< 0.001**		
CRP elevation	1.28 (1.07–1.54)	**0.008**		
Hypokalemia	1.01 (0.77–1.32)	0.958		
Hyperkalemia	1.22 (1.01–1.48)	**0.044**		
Hyponatremia	1.24 (1.03–1.49)	**0.025**		
Hypernatremia	1.59 (1.22–2.07)	**0.001**	1.55 (1.18–2.03)	0.002

*CI* confidence interval, *CRP* C-reactive protein, *HR* hazard ratio, *PT/INR* prothrombin time/international normalized ratio

^a^Based on significant variables (*P* < 0.05) in univariable analysis

## Discussion

The findings of the present study indicate that the prognosis is demonstrably poor for terminally ill cancer patients with hypernatremia (vs. eunatremia or hyponatremia). These results are in accordance with outcomes of previous studies, offering added support. Based on a group of 259 cancer patients referred for palliative care, Alsirafy et al. reported shorter median survival (8 days) and higher mortality (68%) in those with hypernatremia than in hyponatremic or eunatremic patients [10]. However, multivariable analysis of well-known prognostic factors in terminal cancer patients had not been carried out. Our data demonstrate that the association between hypernatremia and poor survival remains robust after controlling for other predictors of survival.

Salahudeen et al. reported poor clinical outcomes involving higher mortality, longer hospitalization, and greater hospital expense in patients with hypernatremia [9]. There were some differences from our cohort of terminal patients whose life expectancies were approximately 6 months. They examined subjects admitted to a comprehensive cancer center with any stage of disease; and their focus was on iatrogenic hypernatremia since baseline hypernatremia contributed so few patients. In our investigation, laboratory testing took place on the day of admission, aimed at existing rather than acquired hypernatremia. Hence, this may be the first effort to explore the prognostic utility of spontaneous hypernatremia in terminally ill cancer patients.

The prevalence of hypernatremia in patients with terminal cancer is unclear. Salahudeen and colleagues found that hypernatremia in cancer patients increased from 0.2% on admission to 2.6% during the course of hospitalization [9]. Another study reported that 8.5% of adult cancer patients referred for palliative care are hypernatremic [10]. Similar to prior studies, we recorded a 3% prevalence of hypernatremia.

Little is known about the specific mechanism by which hypernatremia worsens survival, but there is at least one plausible explanation. Hypernatremia is generally induced by the loss of electrolyte-free water. Physiologic feedback mechanisms, such as thirst and ADH release, are then promptly activated to increase water intake and minimize additional free water loss. In a healthy population, elevated serum sodium levels return to the normal range [12]. However, feedback dysfunction in patients with terminal cancer may impede or prevent normalization of serum sodium concentrations, and many are faced with non-replenishment of water lost through excessive sweating, vomiting, diarrhea, and nasogastric drainage [15]. Impaired response to thirst due to diminished mental faculties or poor oral intake and subsequent dehydration may induce hypernatremia in such patients outside hospital environments. Still, there is virtually no research on hypernatremia in cancer patients. Retrospective studies of older adult patients and critically ill patients admitted to intensive care units would be helpful to understand this problem in the context of terminal cancer [16, 17]. Mental debilitation and poor oral intake create rapid declines in their general conditions [18]. Although causality between hypernatremia and deteriorating general conditions remain in question, hypernatremic patients are extremely ill and less inclined to survive.

Certain prognostic factors—namely poor functional status, leukocytosis, and hypoalbuminemia—are well documented in past reports, but the data on effects of hyponatremia have been inconsistent. Several earlier endeavors have shown the negative prognostic aspect of hyponatremia in a variety of cancers [19, 20]. Yoon et al. also demonstrated a relationship between hyponatremia and shorter survival time in terminally ill cancer patients [21]. However, the results of another study failed to support this relationship in Korean patients with terminal cancer entering a hospice unit, although hampered by a relatively short median survival time (9.5 days) [22]. To our knowledge, the present analysis offers the most fully controlled results in a comparable setting, adjusted for potential confounders. Also, our subjects survived longer than those in previous studies. Even so, hyponatremia was not predictive of poor survival under these conditions. Further prospective studies to explore the prognostic significance of hyponatremia are nevertheless warranted.

At present, evidence of the prognostic value of potassium disorders is sparse. Cui et al. observed an association between serum potassium level and survival time, but significance was not reached in multivariable analysis [23]. In our study, hyperkalemia similarly did not emerge from multivariable analysis as a significant predictor of poor prognosis. One retrospective study conducted in Taiwan cited a serum potassium level > 5 mg/dL as an objective index of short-term survival (7 days) in patients with advanced cancer [11]. However, other researchers found no prognostic significance attached to potassium imbalance [2, 22].

There are acknowledged limitations to interpreting our results. First, the cohort is only representative of a single center, and the number of hypernatremic patients was small. Considering the low prevalence of hypernatremia in terminal cancer patients, large-scale multicenter investigations would be helpful to determine the actual prognostic import of hypernatremia under such circumstances. Another drawback is the lack of sequential or interventional data on serum sodium levels. A previous study indicated that hyponatremia normalization is prognostically beneficial in patients with advanced non-small cell lung cancers [24]. One may thus infer that without normalization, patients with persistent hypernatremia will fare worse. Finally, we did not consider potential confounders related with symptoms such as anorexia-cachexia, delirium, dyspnea, and edema.

## Conclusion

Hypernatremia on admission for palliative care of terminal cancer is predictive of shorter patient survival. Despite its low prevalence (3%), greater clinical attention should be paid to the prognostic utility of hypernatremia.

## Data Availability

The datasets used and/or analyzed during the current study are available from the corresponding author on reasonable request.

## Abbreviations

CRP: C-Reactive Protein
LDH: Lactate DeHydrogenase
INR: International Normalized Ratio
ECOG-PS: Eastern Cooperative Oncology Group Perfomance Status
WBC: White Blood Cell
ADH: AntiDiuretic Hormone
